# Establishment of fracture blister model and analysis of plasma protein markers in rats

**DOI:** 10.3389/fimmu.2025.1547491

**Published:** 2025-04-14

**Authors:** Xin Hu, Peiyuan Wang, Tao Wang, Jingcheng Cao, Kezheng Du, Marius M. Scarlat, Lin Liu, Yutong Li, Xin Wang, Haofei Wang, Huijie Ma, Ling Wang, Lin Jin, Zhiyong Hou

**Affiliations:** ^1^ Department of Orthopaedic Surgery, Hebei Medical University Third Hospital, Shijiazhuang, Hebei, China; ^2^ Key Laboratory of Biomechanics of Hebei Province, Shijiazhuang, Hebei, China; ^3^ University of Timișoara, Timisoara, Romania; ^4^ Baoding University, Baoding, Hebei, China; ^5^ The Key Laboratory of Neural and Vascular Biology, Ministry of Education, Hebei Medical University, Shijiazhuang, Hebei, China; ^6^ Engineering Research Center of Orthopedic Minimally Invasive Intelligent Equipment, Ministry of Education, Shijiazhuang, China; ^7^ National Health Commission (NHC) Key Laboratory of Intelligent Orthopaedic Equipment, Shijiazhuang, Hebei, China

**Keywords:** fracture blister, animal models, cytokines, differentially expressed proteins, plasma protein

## Abstract

**Purpose:**

Fracture blister (FB) is a frequent complication in orthopedic surgery. The primary objective of this study was to refine the animal model of FB and to identify plasma protein markers associated with its development and progression.

**Methods:**

In this study, Sprague-Dawley (SD) rats were used as experimental subjects. Various pressures and compression durations were applied to the lower limbs of rats with fractures to compare the differential expression patterns (DEPs) between the pressure-time combination that resulted in the highest incidence of blisters and other groups. Subsequently, we investigated the variations in DEPs expression across different time intervals of the established model.

**Results:**

Our findings indicate that following a lower limb fracture in SD rats, the highest incidence of blister formation was observed under conditions of 450 mmHg pressure and 9 hours of compression (46%, 7/15). In this group, the levels of CD44 and B2M were significantly elevated, while those of Activin R2A were reduced. Furthermore, we investigated the temporal profile of the group with the highest incidence of blister formation and found that CXCL16 and ROBO1 reached peak secretion 48 hours post-injury, followed by a subsequent decline. Additionally, the secretion of IL-2RG and IL-7 continued to increase 48 hours after the injury.

**Conclusions:**

the increase of CD44 and B2M and the decrease of Activin R2A might be the potential influencing factors for the higher incidence of fracture blisters. CXCL16 and ROBO1 reached their peak 48 hours after the end of molding, and IL-2 RG and IL-7 R continued to increase 48 hours after the end of molding, which will provide a new direction for the study of the occurrence and development mechanism of fracture blisters.

## Introduction

Fractures blister (FB) have an incidence rate of approximately 10.63% in lower limb fractures and are a common complication among patients with lower limb fractures (1). They manifest as large blisters filled with clear fluid or blood. This is because after a fracture occurs, the permeability of the surrounding blood vessel walls increases, and tissue fluid enters the tissue space, further developing into swelling. They typically appear 24 hours after the fracture, but may also emerge as early as 6 hours or be delayed until 72 hours (2). FB appear to be an unwanted phenomenon and might signify complications like infection and soft tissue injury. Nevertheless, some current studies indicate that for certain patients with acute compartment syndrome (ACS), the formation of fracture blisters (FBs) may relieve the pressure in the fascial compartment, which can ease the compression of deep soft tissue and improve the patients’ clinical symptoms (1, 3, 4). This implies that fracture tension blisters may have a positive aspect, and there is a scarcity of research on potential biological markers for blisters in patients with lower-limb fractures.

Owing to the constraints inherent in clinical research, animal models represent indispensable research apparatuses. Our research team has contrived a lower limb fracture model for SD rats (5). In this model, the limb of the rat is fractured via the application of a heavy object in free fall, and then a specific pressure is imposed and sustained for a defined duration. This model is capable of emulating the scenario of elevated pressure within the fascial compartment subsequent to lower limb fractures and muscle trauma, and is consequently also applicable in the context of the acute compartment syndrome (ACS) model. Nevertheless, this model has limitations, as it fails to account for the statistical occurrence of FB. Hence, it is imperative to refine and optimize this model to maximize the probability of FB manifestation to the greatest extent possible.

Inflammation is among the main factors promoting the progression of FB (6). However, at present, there are few blood-related biological markers associated with animal FB, and the relevant indicators are lacking. Over the past decades, High-throughput protein array analysis technology has gradually matured. Protein detection via protein-protein interaction and small-molecule-protein interaction has played an important role in an increasing number of fields, particularly in disease diagnosis and the identification of key factors in normal and diseased processes (7–9). Compared with ELISA technology, it has the characteristics of high throughput and can detect a large number of target factors simultaneously. It also has high specificity and sensitivity (10).

The aim of this study was to seek potential biological markers associated with the occurrence of FB. This was achieved by detecting the blood indices of rat lower limb fracture models with varying probabilities of blisters and comparing the differentially expressed proteins (DEPs) among groups with different blister probabilities using high throughput protein arrays.

## Materials and methods

### Animals and ethics statement

This study was approved by the Ethics Committee of the Third Hospital of Hebei Medical University (approval number: KSD2025-044-1). Male Sprague-Dawley rats aged 12 weeks, weighing between 290-310g, were used. Prior to the experiment, the rats were fed a standard diet and reared in an environment with a 12 - hour light/12 - hour dark cycle, while the temperature (22°C -24°C) and humidity (50%-60%) were controlled.

### Animal modeling

**Figure 1:**
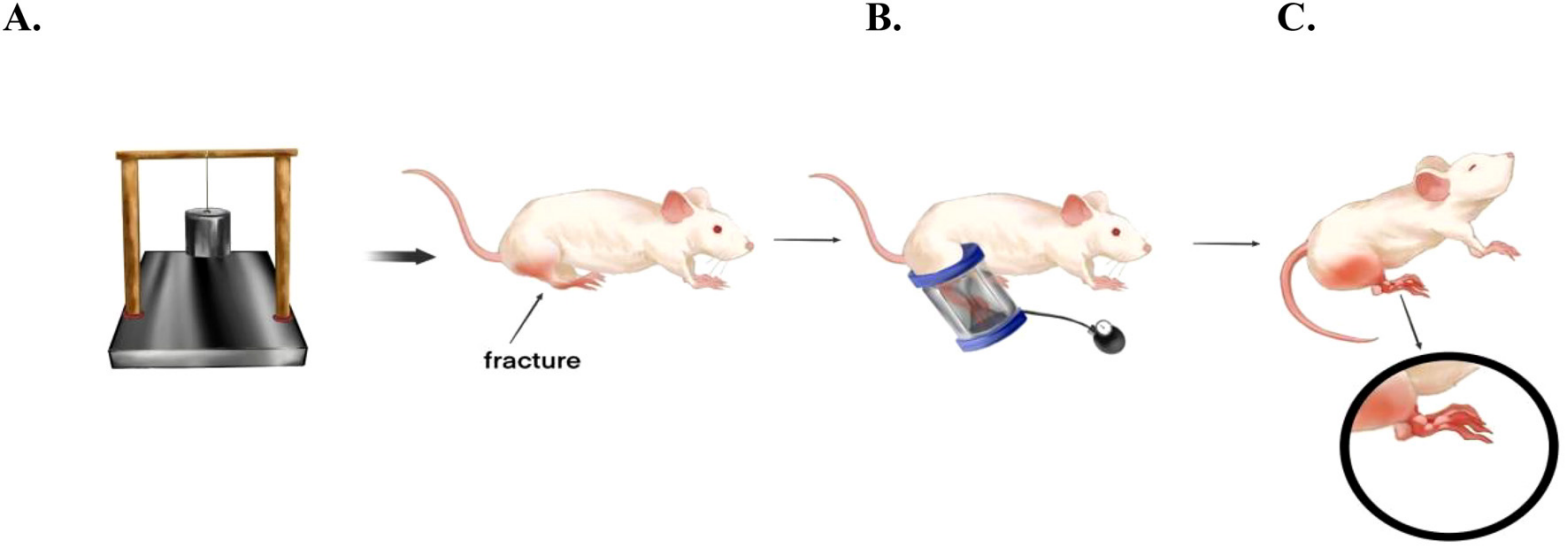
Establishment of the blister model after fracture in rats. **(A)** A device similar to the “guillotine” was utilized, and a weight of 1 kg was freely dropped onto the calf of the rat from a height of 40 cm, causing a fracture of the calf and blunt soft tissue trauma. **(B)** Three hours after the trauma, pressure was applied to the lower leg using a pressure device consisting of a rigid plastic tube embedded with a pressure gauge cuff. The specific pressure and pressure time were determined by the experimental group. **(C)** After completion, the probability of fracture blisters was calculated.

Prior to conducting the experiment, the rats were anesthetized using a 1% concentration of pentobarbital sodium (0.3ml/kg) to guarantee their comfort and absence of pain throughout the procedure. We employed a previously established and validated rat ACS model, involving the use of a 1kg weight that was allowed to fall freely onto the rat’s calf from a height of 40cm, inducing leg fractures and soft tissue blunt trauma. Subsequently, at three hours post-trauma induction, a novel compression device, comprising a pressure gauge cuff (80*8.5mm, supplied by Constant Hui Medical Instruments LTD) embedded within a rigid acrylic plastic tube, was utilized to apply pressure to the rat’s calf via the cuff. The specific pressure levels and durations applied were dictated by the varying experimental groups (**Figure 1**).

### Look for conditions with the highest blister rate

We designed different combinations of pressure (350 mmHg, 400 mmHg, 450 mmHg, and 500 mmHg) and time (6 h, 9 h, 12 h). There were a total of 12 combinations in the groups (**Table 1**). 180 rats were randomly assigned to different groups, with 15 rats in each group. The model was constructed according to the above method. After the model was made, we observed the incidence probability of tension blisters in rats with lower - extremity fractures 24 h later.

**Table 1:** The probability of fracture blisters was calculated by applying different pressures and time to the lower extremities of rats after fracture.

Blister rate	350mmHg	400mmHg	450 mmHg	500 mmHg
6h	13.3% (2/15)	20% (3/15)	33.3% (5/15)	26.7% (4/15)
9h	13.3% (2/15)	33.3% (5/15)	46% (7/15)	33.3% (5/15)
12h	20% (3/15)	26.7% (4/15)	26.7% (4/15)	20% (3/15)

### Plasma collection

After determining the pressure - time combination with the highest probability of blister formation, another 27 rats were prepared. These rats were randomly divided into the experimental groups: ①6 h, 400 mmHg; ②9 h, 400 mmHg; ③12 h, 400 mmHg; ④6 h, 450 mmHg; ⑤9 h, 450 mmHg; ⑥12 h, 450 mmHg; ⑦6 h, 500 mmHg; ⑧9 h, 500 mmHg; ⑨12 h, 500 mmHg, with 3 rats in each group. Blood was collected 24 hours after the molding process. For the optimal combination in terms of blister incidence, an additional 9 rats were prepared, and plasma samples were collected at 2 days, 3 days, and 7 days after the completion of the modeling. Meanwhile, 6 normal rat plasma samples were also taken as the experimental control group.

The blood collection method employed is blood sampling from the cardiac apex. After inducing anesthesia with 3% isoflurane in rats, anesthesia was maintained with 1.5% isoflurane. As much blood as possible was collected from the cardiac apex using EDTA anticoagulant tubes. The blood was mixed thoroughly and allowed to stand for five minutes before being centrifuged at 3000 × 133 rpm for 5 minutes to extract the plasma, which was then stored in a -80°C freezer for future use. Following the blood collection, the rats were euthanized by excessive carbon dioxide inhalation. Upon confirming the death of the animals, their carcasses were incinerated for harmless disposal.

### Protein array detection

The collected plasma from each group of rats was subjected to the detection of 282 antigens using the Ray Bio L-Series Mouse Antibody Array (Ray Biotech, Catalog No. GSR-CAA-282), following the manufacturer’s instructions. Data analysis was performed using the data analysis software specific to GSR-CAA-282.

### Statistical analysis

All data were analyzed and visualized using Graph Pad Prism 9, Microsoft Excel, Cytoscape, and R programming language. Gene sets were created using Gene Ontology (GO) terms and Kyoto Encyclopedia of Genes and Genomes (KEGG) pathways. Differences in proteins between different groups were assessed using the T-test, while comparisons among multiple groups were conducted using One-way ANOVA analysis. A P-value ≤ 0.05 was considered statistically significant.

## Results

### Determination of the pressure-time combination with the highest probability of blistering

Through experimental observation, we discovered that subjecting rats to 9 hours of compression under a pressure of 450 mmHg (group 5) resulted in a significantly higher incidence of blisters compared to other pressure-time combinations, with a blistering rate of 46% (7 out of 15 rats) (**Table 1**). Based on this finding, we established eight additional condition groups (ranging from group 1 to group 9) that mirrored this specific condition and collected plasma samples from them for subsequent protein analysis.

### Multifactorial analysis: comparison between different pressure and time groups

**Figure 2:**
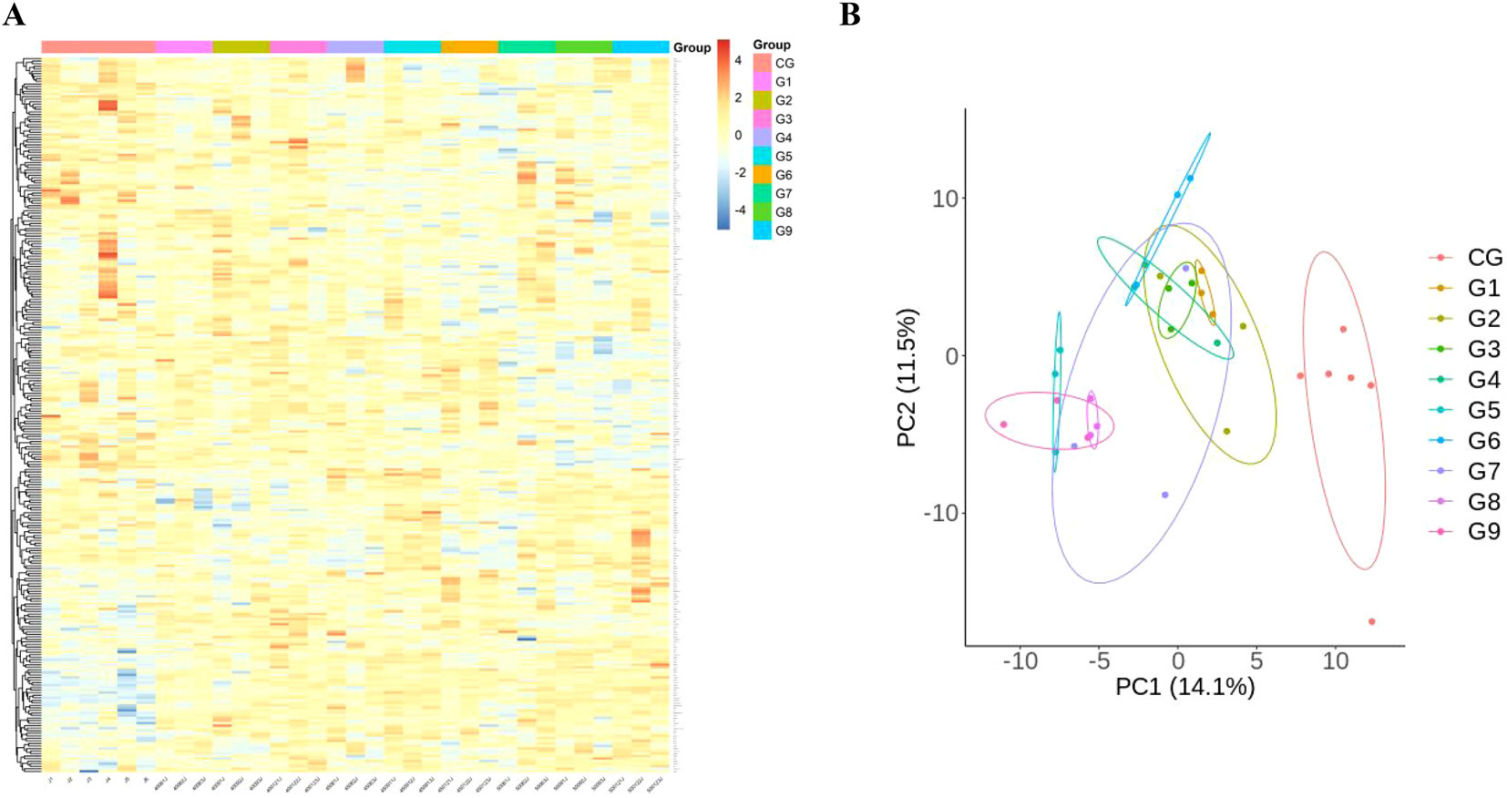
Comparison of immunological proteins under different pressure and time combinations (group 1-9)and control group(group 10). **(A)** Heat map of protein expression among different groups. The x-axis represents the sample group and the y-axis represents the protein name. Different colors signify different levels of protein expression, with blue to red indicating low to high levels of expression. **(B)** Principal component analysis (PCA) reveals the color distinction among different groups. Each dot represents a single experimental rat, with those having similar protein expression profiles located in proximity.

To detect DEPs among different groups, One-way Anova analysis was used to compare the groups, and heat maps and PCA analysis showed differences in protein expression among the 10 groups (**Figures 2A, B**).

**Figure 3:**
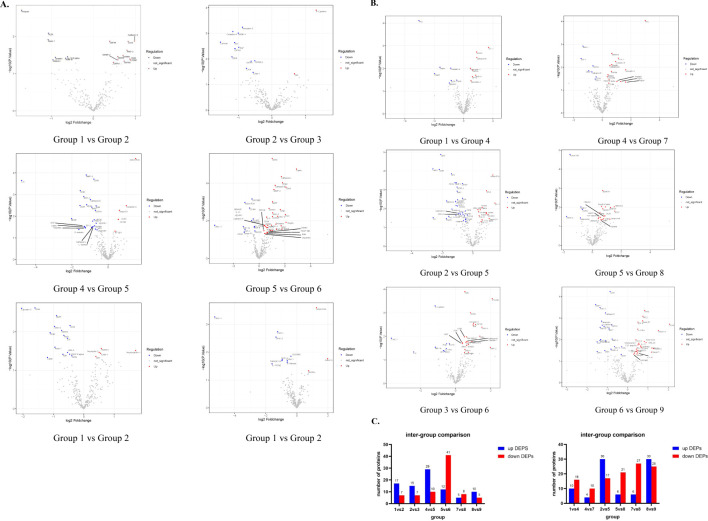
Expression of DEPs between different groups. **(A)** These Volcano maps illustrate the variations in DEPs from 6h to 9h and 9h to 12h when the control pressure conditions (400mmHg, 450mmHg, 500mmHg) remain constant **(B)** These volcano maps demonstrate variations in DEPs ranging from 400 mmHg to 450 mmHg and from 450 mmHg to 500 mmHg while keeping the control time conditions (6h, 9h, 12h) constant. **(C)** The bar chart shows the specific number of up-regulated and down-regulated proteins in DEPs compared between groups.

When the pressure is constant, make pairwise comparisons according to different times. It was discovered that, under constant pressure of 400mmHg, there were 24 DEPs observed in a duration of 9 hours compared to 6 hours (group 1 vs group 2), and 22 DEPs in 12 hours compared to 9 hours (group 2 vs group 3). Notably, 6 of these DEPs remained consistent throughout. When the pressure remained constant at 450mmHg, there were 39 DEPs observed in a duration of 9 hours compared to 6 hours (group 4 vs group 5), and 53 DEPs in 12 hours compared to 9 hours (group 5 vs group 6). Notably, 27 of these DEPs were consistent between the two groups. When the pressure was stabilized at 500 mmHg, there were 13 differentially expressed proteins (DEPs) for the comparison between 9 and 6 hours (group 7 vs group 8), and 15 DEPs for the comparison between 12 and 9 hours (group 8 vs group 9). Among them, only 1 case of DEPs was common (**Figures 3A, C**).

Next, the pairwise comparison of different pressures was carried out, and it was found that when the 6h time was constant, there were 26 DEPs compared with 450mmHg and 400mmHg (group 1 vs group 4). There were 14 DEPs in 500mmHg compared with 450 MMHG (group 4 vs group 7). 2 are consistent. At a constant time of 9h, there were 47 DEPs in 450mmHg compared with 400mmHg (group 2 vs group 5), and 27 DEPs in 12h compared with 9h (group 5 vs group 8), with 7 DEPs consistent between the two groups. At a constant time of 12h, there were 33 DEPs compared with 450mmHg (group 3 vs group 6) and 54 DEPs compared with 9h (group 6 vs group 9), and 9 DEPs were consistent between the 2 groups (**Figures 3B, C**).

The same protein among the DEPs compared in the aforementioned groups was screened. We discovered that when the pressure remained constant at 400 mmHg, the concentration of 2 proteins (FGF-12, Noggin) peaked at 9h, and the concentration of 4 proteins (Cadherin-4, Neuropilin-2, CDNF, CA14) reached the lowest point. When the pressure is sustained at 450 mmHg, the concentrations of 20 proteins (B2M, CD44,B7-2, IL-2, MMP-2, Cathepsin E, etc.) peak at 9 hours, and the concentrations of 7 proteins (Activin R2A, TGF-beta, RIII, TIMP-2, etc.) bottom out. Whereas when the pressure is set at 500 mmHg, only 1 protein (IL-11 RA) reaches its trough concentration at 9 hours. Subsequently, we maintained a constant time and conducted comparisons among different pressures. We discovered that when the time was fixed at 6 hours and the pressure was 450 mmHg, one protein (IGF-1) reached its peak concentration, while one protein (Contactin-4) reached its trough. When the time was fixed at 9 hours and the pressure was 450 mmHg, five proteins (CD44, B2M, HPX, Matrilin-4, MESDC2, and) reached their peak concentrations, while one protein (Activin R2A) reached its trough, and one protein (CD5) exhibited a progressive decrease with the increase in pressure. When the time was fixed at 12 hours and the pressure was 450 mmHg, seven proteins (Cadherin-4, Fractalkine, CD34, B7-2, CNTN, etc.) reached their trough concentrations, one protein (APOH) showed a progressive increase with the increase in pressure, and 1 protein (SIRP alpha) showed a progressive decrease (**Figures 3A, B**). By screening DEPs, we discovered that two proteins, B2M and CD44, were significantly elevated in the group with the highest vesiculation probability (group 5), while one protein, Activin R2A, was significantly reduced, irrespective of the received pressure change and pressure duration.

**Figure 4:**
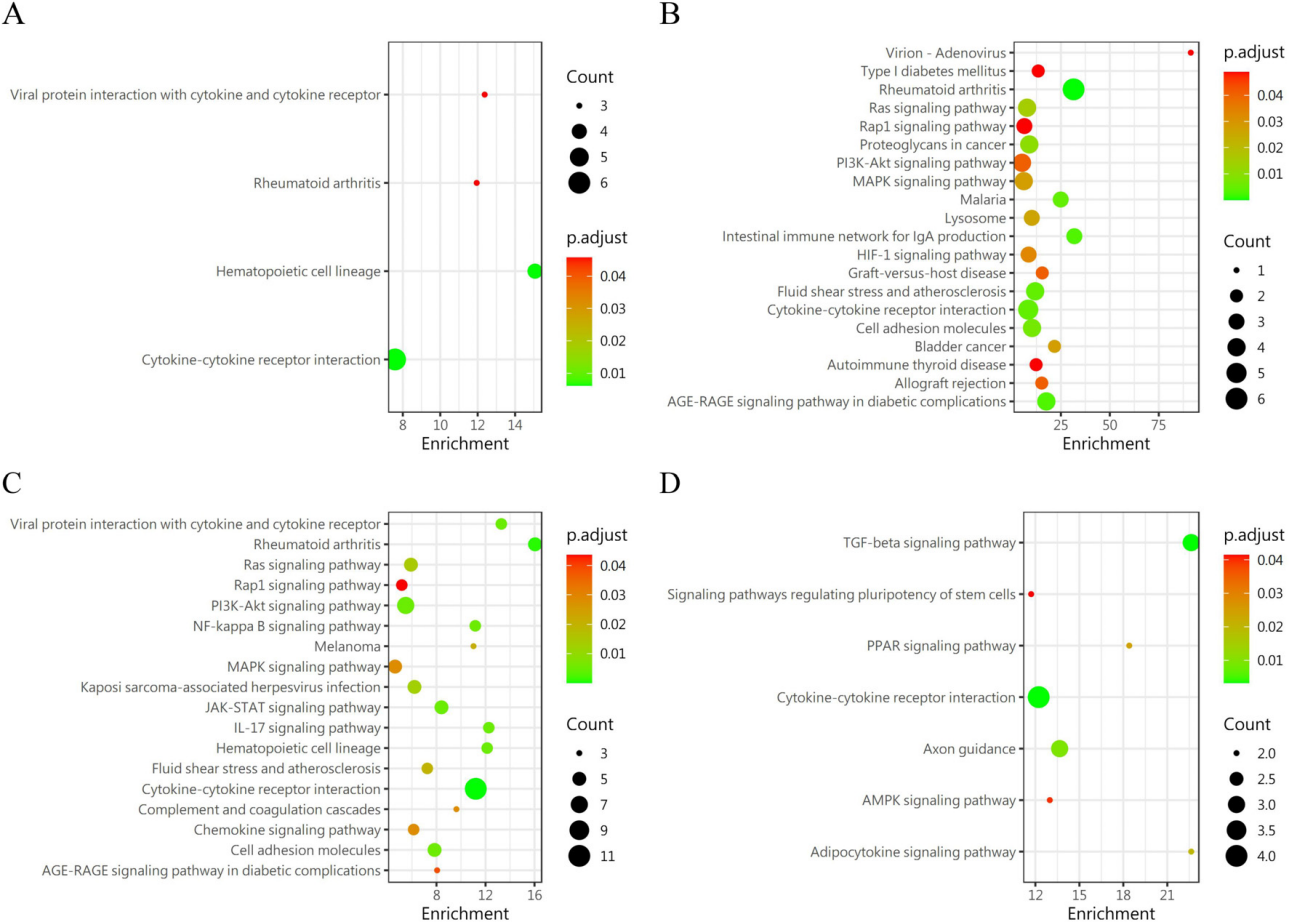
KEGG enrichment analysis of DEPs in the similar condition group as compared with group 5. **(A)** Group 2 vs Group 5 **(B)** Group 4 vs Group 5 **(C)** Group 6 vs Group 5 **(D)** Group 8 vs Group 5.

**Figure 5:**
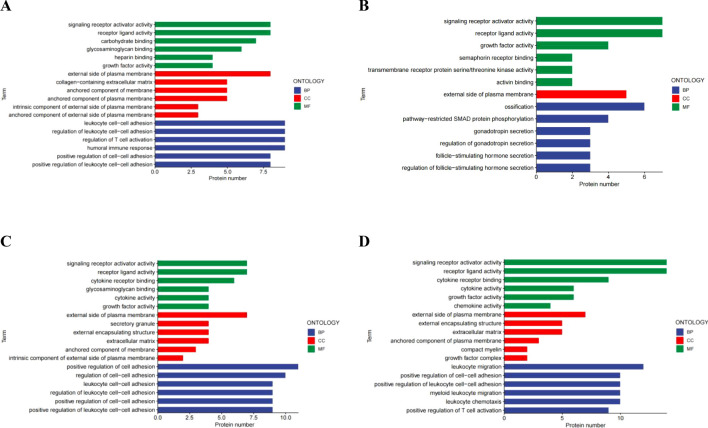
Gene ontology (GO) indicated that the DEPS-related pathways were significantly expressed in group 5 compared to the other groups. BP; Biological Process. CC, Cellular Component; MF, Molecular Function. **(A)** Group 2 vs Group 5 **(B)** Group 4 vs Group 5 **(C)** Group 6 vs Group 5 **(D)** Group 8 vs Group 5.

We carried out a comparative analysis of differential proteins between the groups similar to group 5 (group 2, group 4, group 6 and group 8) and group 5, Meanwhile, it was compared with the control group (group 13). Then, we analyzed the variations of differential proteins in the group 5 through GO (**Figure 4**) and KEGG (**Figure 5**) protein enrichment. KEGG analysis unveiled the aggregations of cytokine interaction, the NF-Kappa b signaling pathway, the Pi3K-Akt cellular pathway, the TGF-beta pathway, and others. GO enrichment analysis showed that Signal receptor activator activity, Receptor ligand activity, external side of plasma membrance, Positive regulation of cell adhesion was obviously enriched. This indicates that the group with the highest probability of blisters showed significant immune and inflammatory responses and tissue repair activity compared with the other groups.

### The group with the highest incidence of blisters demonstrated a time-gradient expression of DEPs

**Figure 6:**
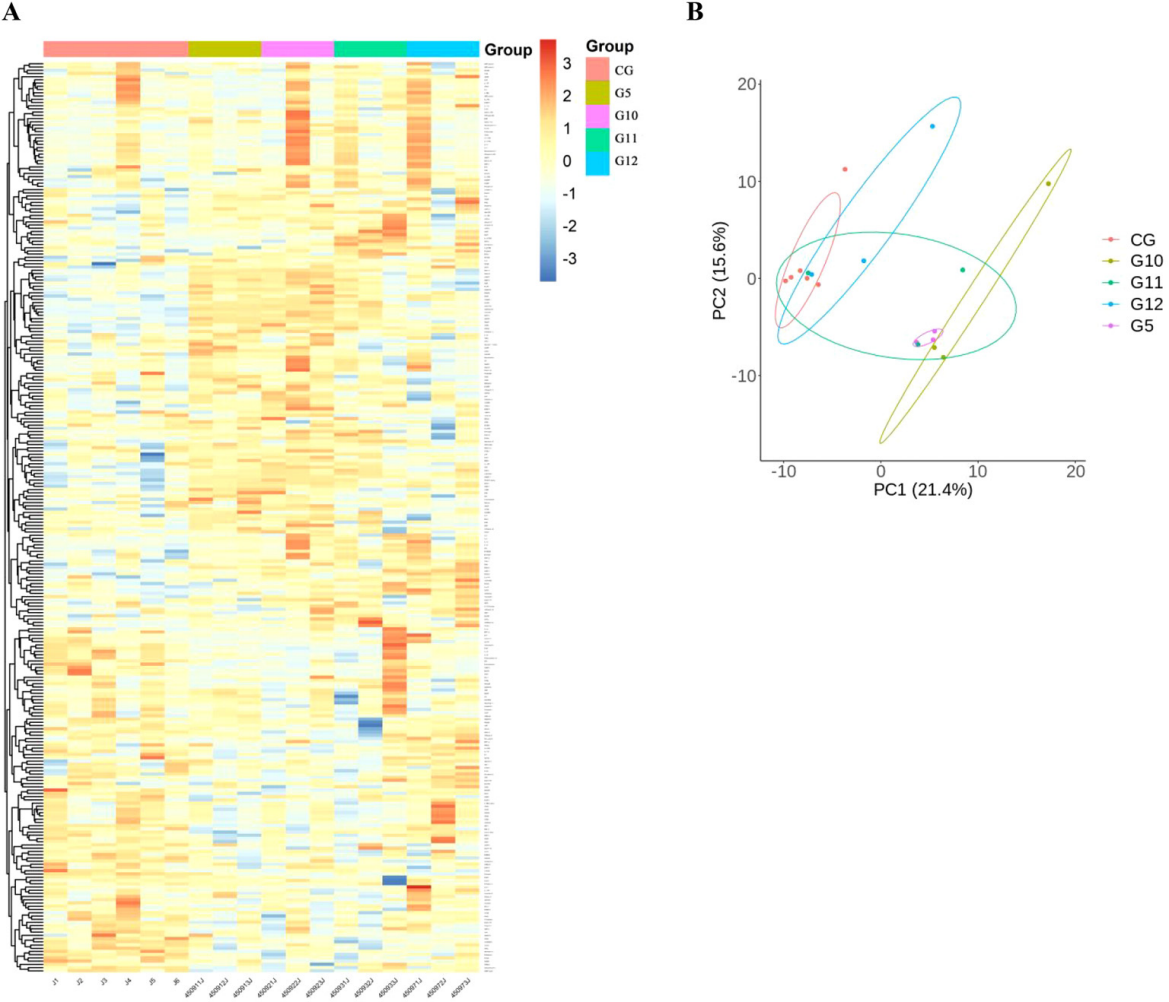
The protein expression of the pressure-time combination (450mmHg,9h) with the highest blister probability was 1day (group 5), 2 days (group 10), 3days (group 11), 7days (group 12) after molding. **(A)** Heat map of protein expression between different groups. The x axis represents the sample group and the y axis represents the protein name. Different colors indicate different levels of protein expression, with blue to red indicating low to high levels of expression. **(B)** Principal component analysis (PCA) revealed color differences between different populations. Each dot represents an experimental rat, with rats with similar protein expression profiles located nearby.

We compared the differences in protein expression among the four time gradients following the modeling of the group with the highest blister rate, namely 1 day, 2 days, 3 days, and 7 days (group 5, group 10, group 11, and group 12), using heatmap and PCA analysis to reveal the differences in protein expression among the ten groups (**Figures 6A, B**).

**Figure 7:**
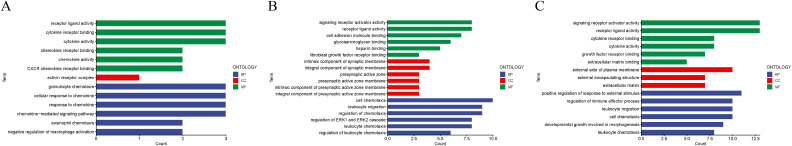
Gene ontology (GO) revealed that the DEPS-related pathways were significantly expressed in group 5 under several distinct time gradients (1day, 2days, 3days, and 7days **(A–C)**).

**Figure 8:**
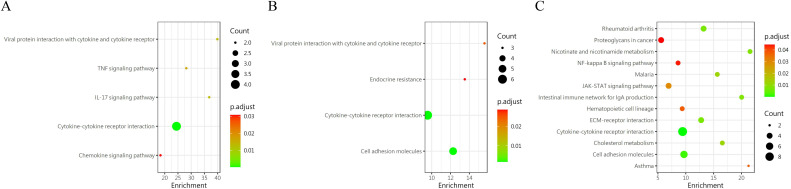
KEGG enrichment analysis of DEP s in group 5 was conducted at different time periods. **(A)** 1 day vs 2 days; **(B)** 2 day vs 3 days; **(C)** 3 day vs 7days.

A pairwise comparison of protein expression across various time points yielded the following observations: At 2 days, compared to 1 day, 5 DEPs were identified, namely CXCL16, TWEAK R, BMP-7, ROBO1, and GDNF. When comparing the protein expression at 3 days to that at 2 days, 24 DEPs were discerned, including IL-13 Ra2, IL-2 RG, NPC2, IL-20 RB, FSTL1, and others. By the 7th day, in comparison to the 3rd day, 7 differential proteins were noted(IL-7 R, NPC2, SEMA7A, LRPAP, EpCAM, IFNA5 and IL-2 RG). Upon further analysis of the data, it was evident that the secretion of two proteins, CXCL16 and ROBO1, peaked at 2 days and subsequently declined after 3 days. Conversely, the secretion of two other proteins, IL-2 RG and IL-7 R, exhibited a continuous increase after 2 days, which persisted until the 7th day, indicating a positive correlation with time. GO (**Figure 7**) reveals intercellular chemokine activity, cytokine-mediated signaling pathways, cytokine activators, and positive regulation of responses to external stimuli. The enrichment of KEGG (**Figure 8**) protein shows the concentration of cell adhesion factors persisted for 2days following the completion of the modeling process, while the concentration of cytokine-cytokine receptor interactions remained elevated for 7 days post-modeling.

## Discussion

This experiment for the first time utilized the probability of blister formation as an observation index by modifying the previously developed rat fracture model and designing different injury models through applying various pressures and durations to the limbs of rats. It was found that by exerting a pressure of 450 mmHg on the lower limbs of rats subsequent to fracturing and subjecting them to a 9-hour compression, the probability of blister formation on the experimental limbs was maximized. Additionally, it was also discovered that the number of differentially expressed proteins in the blister group was significantly greater than that in other groups under similar conditions. The KEGG and GO results reveal that the Blister group presents distinct inflammatory and immune responses, thereby suggesting that the generation of water blisters in patients with lower limb fractures might involve potential reactions, offering valuable references for the research on the mechanism of water blister formation in lower limb fracture patients.

The perspective holds that blister formation constitutes a specific signaling pathway, triggered by the abnormal activation of kinase signaling pathways such as p38MAPK, PKC, and EGFR, giving rise to the reorganization of the cytoskeleton and the disruption of cell connections (11). The emergence of blisters is regarded as a manifestation of skin injury, which may result in an increased risk of infection, prolonged surgical waiting periods, and other issues. Hence, tension blisters are typically considered a complication associated with fractures (12–14). However, our team’s previous discoveries regarding the cytokine pattern of vesicular fluid in patients with cupping, burns, and lower limb fractures have indicated that the expression of cytokines in fracture blisters is not precisely the same as in the other two types of blisters (6). Simultaneously, we also discovered that the occurrence of fracture blisters can lower the internal fascial pressure of the fractured limb, thereby reducing the likelihood of the occurrence of osteofascial compartment syndrome. Therefore, we hypothesize that the emergence of tension blisters in patients with lower limb fractures is a means of releasing pressure within the fascial compartment (3, 4, 15). To test this hypothesis, in animal models, it is necessary to design a model that can generate as many fracture tension blisters as possible, even though the probability of blisters in this injury model is not 100%.

The fracture models of the lower extremity typically comprise closed fracture models and open fracture models. Handoo (16) used Modified three-point bending pliers as a device to create the closed rat tibial bone fracture; Chen (17) established the fracture model by incising the lower extremity skin and performing osteotomy. Doğan (18) modeled the fracture by dropping a heavy object from a height in a “guillotine” fashion to disrupt the integrity of the lower limb bone. Our team has formerly designed an ACS model for the lower limbs of rats, which involves applying 300 mmHg pressure to the lower legs of rats for 6 hours after the lower legs of rats were fractured by a fall from a height with a 1 Kg weight (5). This animal model is characterized by the combination of blunt trauma and crush injury, thereby better simulating the occurrence of FB. The weight drop is employed to simulate the condition of patients with lower limb fractures, and pressure is applied to mimic the tight situation around the lower leg compartment and restrict the diffusion of pressure within the fascia. Since the skin of the lower limbs in rats is looser than that in pigs, dogs, and other animals, pressure is prone to spread. The model also embodies the classic diagnostic criteria of ACS, encompassing ICP, ΔP, plasma biochemistry, pathology, and changes in lower limb blood flow (19, 20).

Through the analysis of the experimental results, it was found that the number of distinct proteins in the group with the greatest number of blisters, in comparison to other conditions, was significantly higher than that between other conditions, suggesting that there is a series of reactions during the emergence of fracture tension blisters. Meanwhile, when comparing the groups, an interesting phenomenon was discovered. When controlling pressure and time, with one condition fixed and the other condition being compared, it is generally believed that the differential proteins therein would continuously change positively or negatively along with the increase of time or pressure. However, we discovered that there were not numerous such differential proteins, and some of the differential proteins would reach a peak or trough value at 9h or 450 mmHg, and subsequently decline/rise at 12h or 500 mmHg. This fully demonstrates that the serological changes in the animal models we designed with the most blistering were significantly distinct from those in other models. We screened these differential proteins and selected those associated with the highest blister rate group, encompassing 25 ascending proteins and 8 descending proteins. Among these, CD44, B2M, and Activin R2A are commonly identified proteins in DEPs. The secretion levels of CD44 and B2M reach their peak values in Group 5, whereas the secretion level of Activin R2A reaches its nadir in Group 5. These observations suggest that these three proteins may play a role in promoting blister formation following a lower limb fracture.

CD44 is a transmembrane glycoprotein that facilitates intercellular adhesion and interaction through its binding to extracellular matrix components such as hyaluronic acid (HA), fibronectin, and osteopontin (21). The actin cytoskeleton is linked via the ERM (ezrin-radixin-moesin) protein family, which plays a crucial role in influencing cell morphology and motility (22). Phosphatidylinositol-4,5-bisphosphate (PIP2) expedites the formation of the CD44-ERM complex, thereby enhancing cell migration. Additionally, the binding affinity between CD44 and HA can activate Rho GTPase, subsequently stimulating the PI3K-Akt signaling pathway. This activation forms a positive feedback loop, which confers resistance to apoptosis and sustains cell survival (23, 24). B2M (β2-microglobulin) is a small protein ubiquitously present in the human body, playing a crucial role in the immune system. It binds to the heavy chains of MHC I (Major Histocompatibility Complex Class I) molecules to form MHC I complexes, which are essential for antigen presentation and recognition (25). B2M is capable of directly modulating the activity of cytokines. Furthermore, B2M interacts with cytokines, including tumor necrosis factor (TNF) and interferon-γ (IFN-γ), to influence their roles in immune responses and immune regulation (26, 27). Activin R2A (Activin Receptor Type IIA), also known as ACVR2A, is a critical transmembrane serine/threonine kinase receptor. As a pivotal component of the Activin signaling pathway, it plays a significant role in regulating cell proliferation, differentiation, apoptosis, and the maintenance of tissue homeostasis (28). Recent studies have demonstrated that Activin R2A is also involved in muscle growth. Notably, research by Lee SJ et al. has shown that the soluble form of ACVR2B exerts a potent enhancing effect on muscle growth (29). Ding H (30) discovered that Activin A, by binding to the Activin R2A receptor, activates the p38β MAPK (mitogen-activated protein kinase) signaling pathway, which results in enhanced degradation of muscle proteins and subsequently leads to muscle atrophy. DEPs analysis revealed that following a lower limb fracture, the increased production of FBs contributes to inflammation and potential tissue repair. The decreased levels of Activin R2A suggest that muscle hyperplasia and muscle atrophy are inhibited. We hypothesize that after a lower limb fracture, the pressure in the osteofascial compartment increases due to inflammation, soft tissue swelling, and hematoma. The presence of FBs aids in reducing this pressure, thereby facilitating the recovery of the injured muscle and surrounding soft tissues.

We performed a DEPs analysis on the time gradient post-molding for the group exhibiting the highest probability of blistering (Group 5). By analyzing four distinct time points—1 day, 2 days, 3 days, and 7 days. we observed that the secretion levels of CXCL16 and ROBO1 proteins peaked at 2 days and subsequently decreased. In contrast, the secretion of IL-2 receptor gamma (IL-2RG) and IL-7 receptor (IL-7R) began to rise 2 days after the completion of the modeling process and continued through to 7 days. The CXCL16, a multifunctional chemokine, exerts dual influences on both inflammatory responses and tumor progression (31, 32), It also contributes to the acceleration of atherosclerotic plaque formation and development (31, 33). Robo1, a transmembrane receptor, plays a crucial role in multiple biological processes. By interacting with Slit proteins, Robo1 directs the oriented growth of axons (34, 35). Additionally, Robo1 inhibits excessive angiogenesis, thereby preserving the normal structure and function of blood vessels (36). IL-2RG serves as a common receptor for multiple cytokines, including IL-2, IL-4, IL-7, IL-9, IL-15, and IL-21. By binding to the specific subunits of these cytokines, IL-2RG facilitates signal transduction, activates the JAK/STAT pathway, and promotes cell survival and proliferation (37). IL-7R is an essential cell surface receptor that mediates multiple signaling pathways by binding to its ligand IL-7, thereby regulating the development, survival, proliferation, and function of T lymphocytes (38, 39). We hypothesized that two days following the cessation of the modeling process, the inflammatory response, cytokine interactions, and immune response in the experimental animals would reach their peak levels. Concurrently, damaged cells would initiate repair and proliferation processes, and T cells and other immune cells would increase in number and become activated, thereby facilitating the recovery of the injured organism.

There are still limitations in this study. First, the number of experimental animal samples is still insufficient, so it is better to increase the number of samples. Secondly, due to the small body size of rats, it is difficult to collect samples in a minimally invasive way due to existing technical problems. Therefore, batch killing can only be adopted when time gradient sampling is performed on the group with the highest blister. If dynamic living sampling can be adopted, it can better reflect the change of DEPs over time.

## Conclusion

By refining a previously established ACS model for SD rats, we developed a novel SD rat model exhibiting a higher incidence of FBs. Concurrently, we identified three proteins associated with the likelihood of FBs occurrence: CD44, B2M, and Activin R2A. Furthermore, we observed that several proteins,CXCL16,ROBO1,IL-2 RG and IL-7, Rexhibited temporal changes following the completion of the modeling process, providing valuable insights into the pathogenesis and progression of FBs.

## Data Availability

The original contributions presented in the study are included in the article/supplementary materials. Further inquiries can be directed to the corresponding authors.
